# Ciprofloxacin and pegylated G-CSF combined therapy mitigates brain hemorrhage and mortality induced by ionizing irradiation

**DOI:** 10.3389/fpubh.2023.1268325

**Published:** 2023-12-14

**Authors:** Juliann G. Kiang, Georgetta Cannon, Matthew G. Olson, Min Zhai, Akeylah K. Woods, Feng Xu, Bin Lin, Xianghong Li, Lisa Hull, Suping Jiang, Mang Xiao

**Affiliations:** ^1^Radiation Combined Injury Program, Department of Scientific Research, Armed Forces Radiobiology Research Institute, Bethesda, MD, United States; ^2^Department of Medicine, Uniformed Services University of the Health Sciences, Bethesda, MD, United States; ^3^Department of Pharmacology and Molecular Therapeutics, Uniformed Services University of the Health Sciences, Bethesda, MD, United States

**Keywords:** mouse, Ciprofloxacin, G-CSF, radiation, platelet, glial fibrillary acidic protein, ICAM1, hemorrhage

## Abstract

**Introduction:**

Brain hemorrhage was found between 13 and 16 days after acute whole-body 9.5 Gy ^60^Co-γ irradiation (IR). This study tested countermeasures mitigating brain hemorrhage and increasing survival from IR. Previously, we found that pegylated G-CSF therapy (PEG) (i.e., Neulasta^®^, an FDA-approved drug) improved survival post-IR by 20–40%. This study investigated whether Ciprofloxacin (CIP) could enhance PEG-induced survival and whether IR-induced brain hemorrhage could be mitigated by PEG alone or combined with CIP.

**Methods:**

B6D2F1 female mice were exposed to ^60^Co-γ-radiation. CIP was fed to mice for 21 days. PEG was injected on days 1, 8, and 15. 30-day survival and weight loss were studied in mice treated with vehicles, CIP, PEG, or PEG + CIP. For the early time point study, blood and sternums on days 2, 4, 9, and 15 and brains on day 15 post-IR were collected. Platelet numbers, brain hemorrhage, and histopathology were analyzed. The cerebellum/pons/medulla oblongata were detected with glial fibrillary acidic protein (GFAP), p53, p16, interleukin-18 (IL-18), ICAM1, Claudin 2, ZO-1, and complement protein 3 (C3).

**Results:**

CIP + PEG enhanced survival after IR by 85% vs. the 30% improvement by PEG alone. IR depleted platelets, which was mitigated by PEG or CIP + PEG. Brain hemorrhage, both surface and intracranial, was observed, whereas the sham mice displayed no hemorrhage. CIP or CIP + PEG significantly mitigated brain hemorrhage. IR reduced GFAP levels that were recovered by CIP or CIP + PEG, but not by PEG alone. IR increased IL-18 levels on day 4 only, which was inhibited by CIP alone, PEG alone, or PEG + CIP. IR increased C3 on day 4 and day 15 and that coincided with the occurrence of brain hemorrhage on day 15. IR increased phosphorylated p53 and p53 levels, which was mitigated by CIP, PEG or PEG + CIP. P16, Claudin 2, and ZO-1 were not altered; ICAM1 was increased.

**Discussion:**

CIP + PEG enhanced survival post-IR more than PEG alone. The Concurrence of brain hemorrhage, C3 increases and p53 activation post-IR suggests their involvement in the IR-induced brain impairment. CIP + PEG effectively mitigated the brain lesions, suggesting effectiveness of CIP + PEG therapy for treating the IR-induced brain hemorrhage by recovering GFAP and platelets and reducing C3 and p53.

## 1 Introduction

It is reported that ~60% of radiation injuries (RI) result from nuclear detonation and accidents (1). Ionizing radiation (IR) results in devastating detriments to radiation-sensitive cells, organs and systems in humans (2–5). The organs and tissues that are known to be acutely sensitive to IR are bone marrow, gastrointestinal tissues, immune cells, spleen, the reproductive system and brain (4). This laboratory reported that irradiation caused brain hemorrhage through the entire brain, both internally and on the surface (6, 7). Many bleeding patches were found in the cerebellum, the medulla oblongata and the pons (7). IR-induced brain hemorrhage was mediated by reductions in both ATP production and platelet counts (7).

The United States Food and Drug Administration (FDA) has approved Neupogen (i.e., G-CSF) and Neulasta (i.e., pegylated G-CSF or PEG) in 2015, Nplate in 2019, and Leukine in 2020 for treating hematopoietic radiation syndrome. They are mainly for bone marrow repair. In a mouse experimental model of radiation, either G-CSF or PEG treatment post irradiation improved mouse survival by 20–40% above the vehicle treatment (8–12). PEG treatment significantly improved bone marrow cellularity and peripheral neutrophil and platelet counts (7, 11).

Due to this limited 20–40% survival improvement through hematopoietic ARS treatment, interventions enhancing the repair of other organs are suggested. Ciprofloxacin (CIP) was selected because it is FDA-approved and listed in the National Strategic Emergency Stock Pile (13). CIP increased human healthy peripheral blood monocytic cells (PBMC) survival from IR (14) and sensitized cancer cells to radiation therapy (14). Additionally, using a high throughput screening approach, CIP was identified as a radioprotector (15). Moreover, CIP + G-CSF has been indicated as a currently recommended standard therapy for hematopoietic acute radiation syndrome (ARS) (13). Therefore, CIP was selected for this study.

ARS contributes to radiation-associated mortality. ARS includes hematopoietic ARS, GI ARS, cutaneous ARS and cranial ARS (1). Among them, cranial ARS has only recently been studied (4).

Radiation induces brain injury (6) and hemorrhage throughout the brain surface and internally (7). In the hindbrain, exposure to radiation resulted in decreased PGC-1α, accompanied by an increase in interleukin (IL)-6, keratinocyte chemoattractant (KC), Eotaxin, granulocyte-colony stimulating actor (G-CSF), macrophage inflammatory protein (MIP)-2, monocyte chemoattractant protein (MCP)-1 and macrophage inflammatory protein (MIP)-1α followed with decreases in IL-2, IL-9. Monokine induced by gamma (MIG) and interferon (IFN)-γ. Additionally, radiation decreased adenosine triphosphate (ATP) production through the inhibition of nuclear respiratory factor (NRF)1, NRF2 p28, and complexes I-V; and an increase in phosphorylation of protein (p) 38 and serine/threonine-specific protein kinase (AKT), and p53 levels along with decreased caspase-3 activation was observed as well (7).

In this study, we investigated whether CIP and PEG combined therapy enhanced survival and mitigated the IR-induced brain hemorrhage. Our hypothesis was that the combined therapy of CIP and PEG induced enhancement of 30-day survival and mitigated brain hemorrhage after lethal IR exposure. Herein, our data demonstrate that IR induced brain hemorrhage and mortality. The increases were mitigated by CIP and PEG combined therapy, thus proving the main hypotheses, CIP enhanced PEG efficacy to improve survival after IR, possibly partly by mitigating brain intracranial hemorrhage through increasing GFAP and decreasing p53 activation.

Our laboratory recently reported that brain hemorrhage was observed on days 13–16 after IR in an experimental animal model of radiation combined with burn trauma (16). In that report, when mice were exposed to 15% total skin surface burn following 9.5 Gy ^60^Cobalt-γ photon radiation, extracranial hemorrhage and intracranial hemorrhage were found. Extracranial hemorrhage was observed in the olfactory lobe, mid-brain, and cerebellum. The latter displayed bleeding that was distributed widely. Histological examination showed subdural and intraparenchymal bleeding in the cerebral cortex and cerebellar cortex. Platelet depletion concurrently occurred, suggesting a correlation between platelet counts and brain hemorrhage (16).

## 2 Materials and methods

### 2.1 Animal and experimental design

Animal procedures were reviewed and approved by the university Institutional Animal Care and Use Committee (IACUC). Euthanasia was performed according to the recommendations and guidance of the American Veterinary Medical Association. The project was carried out in the university facility accredited by the Association for Assessment and Accreditation of Laboratory Animal Care (AAALAC).

B6D2F1/J female mice (12 weeks old, ~20–26 g) purchased from Jackson Laboratory (Bar Harbor, ME) were maintained in a facility accredited by AAALAC in plastic microisolator cages with hardwood chip bedding and allowed to acclimate to their surroundings for at least 3 days prior to initiation of the study. Male mice were not used in this study because of potential problems associated with male mouse aggression, such as fight wounds which were not desirable during the experimental period. Previous injury studies (10, 11, 16–18) also used female mice for this reason. As such, we continued to conduct this study with female mice so that data collected could be compared with previous ones.

These mice were provided with commercial rodent chow (Rodent Diet #8604, Harlan Teklad, Madison, WI) and acidified tap water (pH = 2.5–2.8) *ad libitum*. Rooms holding animals were maintained at 22°C ± 2°C with 50 ± 20% relative humidity using at least 10–15 air changes/h of 100% conditioned fresh air with a 12-h 0600 (light) to 1800 (dark) full-spectrum lighting cycle. Mouse tails were tattooed for individual identification during acclimation.

B6D2F1/J female mice were randomly divided into 8 groups: (1) sham + vehicle for PEG (V1) + vehicle for CIP (V2); (2) IR + V1 + V2; (3) sham + CIP + V1; (4) IR + PEG + Veh2; (5) sham + V2 + PEG; (6) IR + V2 + PEG; (7) sham + CIP + PEG; and 8) IR + CIP + PEG. The sham-irradiated animals (equivalent to 0 Gy) were treated in the same manner as the irradiated animals but not exposed to the radiation source. For the 30-day survival study, *N* = 20 per group. For early time point studies, *N* = 6 per group.

### 2.2 Gamma irradiation

Mice were given 9.5 Gy (10, 11, 16–18) whole-body bilateral ^60^Co γ-photon radiation at AFRRI high-level Co-60 facility (Nordion Inc, Ottawa, Canada), delivered approximately at dose rate of 0.4 Gy/min, as described previously (18). The dose of 9.5 Gy is expected to cause the death of 50% of the population over 30 days post irradiation, abbreviated LD_50/30_. The field was uniform within ±2%. The exposure time for each radiation was determined from the mapping data; corrections for the ^60^Co decay and the small difference in the mass energy absorption coefficients for water and soft tissue were applied. The accuracy of the actual dose delivery was verified with an ionization chamber (Exradin A12, Standard Imaging, Madison, WI) adjacent to the mouse rack, which had been calibrated in terms of dose to the midline soft tissue of mice.

### 2.3 Ciprofloxacin administration

CIP (NDC: 63739-700-10) was purchased from Aurobindo Pharma Limited (Hyderabad, India). For the 30-day survival study, CIP at 90 mg/kg was orally administered 2 h after irradiation and thereafter once daily for 21 days after irradiation. For the early time-point studies, CIP was orally administered in the same manner but for fewer days (up to only 14 days). The vehicle given to sham mice was drinking water (18).

### 2.4 Pegylated G-CSF administration

Pegylated G-CSF (Neulasta^®^ NDC: 555-13-019001, PEG) is a polyethylene glycol pharmaceutical-formulated-grade drug, also known as pegfilgrastim, that was purchased from the AmerisourceBergen Corporation (Valley Forge, PA). For the 30-day survival study, PEG at a dose of 1000 μg/kg was administered by s.c. injections (10, 11) in a volume of 0.2 ml at 24 h, day 8, and day 15 after irradiation, i.e., 25 μg/25-g mouse. For the early time-point study, PEG at the same dose was s.c. injected at 24h, day 8, and day 14 in order to meet the scheduled blood/tissue collection on days 2, 4, 9 and 15. PEG was supplied in 0.6 mL prefilled syringes for s.c. injection. Each syringe contained 6 mg PEG in a sterile, clear, colorless, preservative-free solution containing 0.35 mg acetate, 0.02 mg polysorbate 20, 0.02 mg sodium, and 30 mg sorbitol in water for injection, USP. The vehicle mouse received 0.2 ml of vehicle containing 0.35 mg acetate, 0.02 mg polysorbate 20, 0.02 mg sodium, and 30 mg sorbitol in 0.6 mL water (10, 11).

### 2.5 Survival

Mice subjected to irradiation were monitored 2–3 times daily for 30 days to determine the survivability under the vehicle control and drug treatment.

#### 2.5.1 Euthanasia

Mice found moribund as described by rodent intervention score sheet (Table 1) and not used for collection of specimens were euthanized by CO_2_ inhalation at a metered fill range of 30–40% followed by cervical dislocation. Terminal CO_2_ euthanasia was used for animals that had successfully survived the experimental procedures, including drug treatment and irradiation.

**Table 1:** Rodent intervention score sheet.

**Parameter**	**Description**	**Score**
Appearance	Normal (coats smooth, eyes/nose clear)	0
	Reduced grooming OR minor hunching	1
	Ocular/nasal discharge AND/OR rough coat and hunching OR facial edema	3
	Emaciated, dehydrated, OR soft stools (fecal matter around anus)	5^*^
	Presence of bloody diarrhea	9
General behavior	Normal	0
	Minor changes – writhe or grimace, slightly less active than baseline	1
	Moderate less mobile and alert	2
	Ataxia, wobbly, appearing weak	6^*^
	Unable to stand	12
Respiratory rate	Normal breathing	0
	Increased (double) breathing rate, rapid or shallow	6
	Abdominal breathing (gasping +/- open mouth breathing)	12
Provoked behavior	Normal	0
	Subdued or weak, but moves away when stimulated	1
	Subdued even when stimulated (moves away slowly)	3
	Unresponsive when stimulated, weak, precomatose	6^*^
	Does not right when placed gently on side within 5 s, or no response when toes pinched -	12

^*^Notify VSD immediately – may need to euthanize. <6 – normal. 6–9 – Morbid, some pain/distress, monitor at least three times a day. >10 – Moribund. Either euthanize or notify VSD. If any single category is at 12, euthanize animal immediately. Mice found to have lost >35% of their body weight (relative to weight at start of experiment) will be brought to the attention of VSD staff or euthanized immediately. VSD, Veterinary Science Department.

### 2.6 Body weight measurement

Body weights of each mouse from all groups during the 30-day survival study period were measured on days 1, 3, 7, 14, 21, and 28 after irradiation.

### 2.7 Blood collection

Mice were anesthetized by isoflurane inhalation at a metered range of 3–5% mixed with 100% oxygen gauged at 500–1000 cc/min in the isoflurane chamber. Then, the anesthetized mouse was moved into the biological hood, placed with its nose to the funnel that was connected to the isoflurane instrument, and blood was collected through cardiac punch. Cervical dislocation was performed to confirm death after blood collection. Partial blood was submitted for platelet counts and the rest of blood was kept at room temperature for 30 min before serum was prepared and stored at −80^o^C until use.

### 2.8 Platelet counts

Blood samples were collected in EDTA tubes and assessed with the ADVIA 2120 Hematology System (Siemens, Deerfield, IL). Differential analysis was conducted using the peroxidase method and the light scattering techniques recommended by the manufacturer.

### 2.9 Brain surface hemorrhage

After mice were anesthetized by isoflurane followed by exsanguination (for blood collection) and cervical dislocation on specified days after sham or irradiation, their entire brains were collected for counting hemorrhage patches on dorsal and ventral surfaces through the entire surface.

### 2.10 Histopathology assessment

After counting the hemorrhage patches on the brain, half of the extracted brains were kept in 10% neutral buffered formalin until processing by routine methods for histopathologic examinations. The formalin-fixed tissues were embedded in paraffin, longitudinally cut into 5-μm sections, stained with hematoxylin and eosin, and examined by light microscopy. The histology slides were scanned using Zeiss Axioscan.Z1. Then, hemorrhage patches were counted through olfactory lobe, forebrain, midbrain and hindbrain, cerebellum, pons, and medulla oblongata, using Zen 2 software (Zeiss Company, Thornwood, NY). The other half brain was stored at −80^o^C until use.

For evaluating bone marrow megakaryocytes, sternums were fixed in 10% neutral buffered formalin and processed with the same procedure as for brain tissues. Likewise, the sternum histology slides were scanned using Zeiss Axioscan.Z1. Then, megakaryocytes on four fields at 40X were counted and averaged as the number for one animal using Zen 2 software.

### 2.11 Tissue lysates

Because the hemorrhagic lesions were dominant in cerebellum, pons and medulla oblongata, this section was used for further biochemical studies. Samples were homogenized using the Bullet Blender Homogenizer Storm (Next Advance, Averill Park, NY) for 4 min at speed 10 in Na ^+^ Hanks' solution containing 10 μl/ml protease inhibitor cocktail, 10 μmol/mL phosphatase 2 inhibitor, 10 μmol/mL phosphatase 3 inhibitor, 10 μmol/mL DTT, 5 μmol/mL EDTA and 10 μmol/mL PMSF. The lysates were centrifuged at 9,000 × g at 4^o^C for 10 min (Sorvall Legend Micro 21 Centrifuge, Thermo Electron Corp, Madison, WI). Supernatant fluids were conserved for protein determination and stored at −80^o^C until use.

### 2.12 Western blot

Total protein in the brain lysates was determined with Bio-Rad reagent (Bio-Rad, Richmond, CA). Samples with 20 μg of protein in Na ^+^ Hanks' buffer containing 1% sodium dodecyl sulfate (SDS) and 1% 2-mercaptoethanol were resolved on SDS-polyacrylamide slab gels (Novex precast 4–20% gel, Invitrogen, Carlsbad, CA). After electrophoresis, proteins were blotted onto a polyvinylidene difluoride (PVDF) membrane (0.45 μm, Invitrogen) using a Trans-Blot Turbo System and the manufacturer's protocol (Bio-Rad, Hercules, CA). The blot was then incubated for 60 min at room temperature with 5% non-fat dried milk in tris-buffered saline-0.5% TWEEN^®^ 20 (TBST) at room temperature. After blocking, the blot was incubated with selected antibodies against GFAP (ABCAM cat# ab7260), Claudin-2 (Invitrogen cat# 32–5600), ICAM1 (ABCAM cat#179707), ZO-1 (Invitrogen cat# 61–7300), p16 (Invitrogen, cat# PA5–20379), p21 (ABCAM cat# ab188224), p53 (Cell Signaling cat# 9282), p-p53 (Santa Cruz, sc-51690), GAPDH (Novus Biologicals, Littleton, CO), and IgG (R & D Systems, Minneapolis, MN) at a final concentration of 1 μg/ml in TBST - 5% milk. The blot was washed 3 times (10 min each) in TBST before incubating for 60 min at room temperature with a 1000X dilution of species-specific IgG peroxidase conjugate (R&D Systems, Minneapolis, MN) in TBST. The blot was washed 6 times (5 min each) in TBST before detection of the peroxidase activity using the Enhanced Chemiluminescence kit (Amersham Life Science Products, Arlington Height, IL). IgG and GAPDH levels were not altered by radiation and were used as controls for protein loading. Pictures of the membranes were taken with a Syngene G:box 9Mp camera using the Gensys program V.1.84.0, and protein bands of interest were quantitated using Genetools program V.4.3.14.0 (all from Synoptics Limited, Cambridge, UK). The bands were normalized to either IgG or GAPDH levels. Data were expressed as the intensity ratio to 0 Gy (the sham + V1 + V2 group) as V1 being vehicle for PEG and V2 being vehicle for CIP.

### 2.13 Statistical analysis

Data were expressed as mean ± S.E.M. For survival, the log-rank test was used for comparison. For each western blot and assay, the data were compared using the ANOVA, Tukey *post-hoc* test, and student's *t*-test with a significance level of 5%.

## 3 Results

### 3.1 CIP enhances PEG efficacy on 30-day survival and mitigates body weight loss after IR

**Figure 1:**
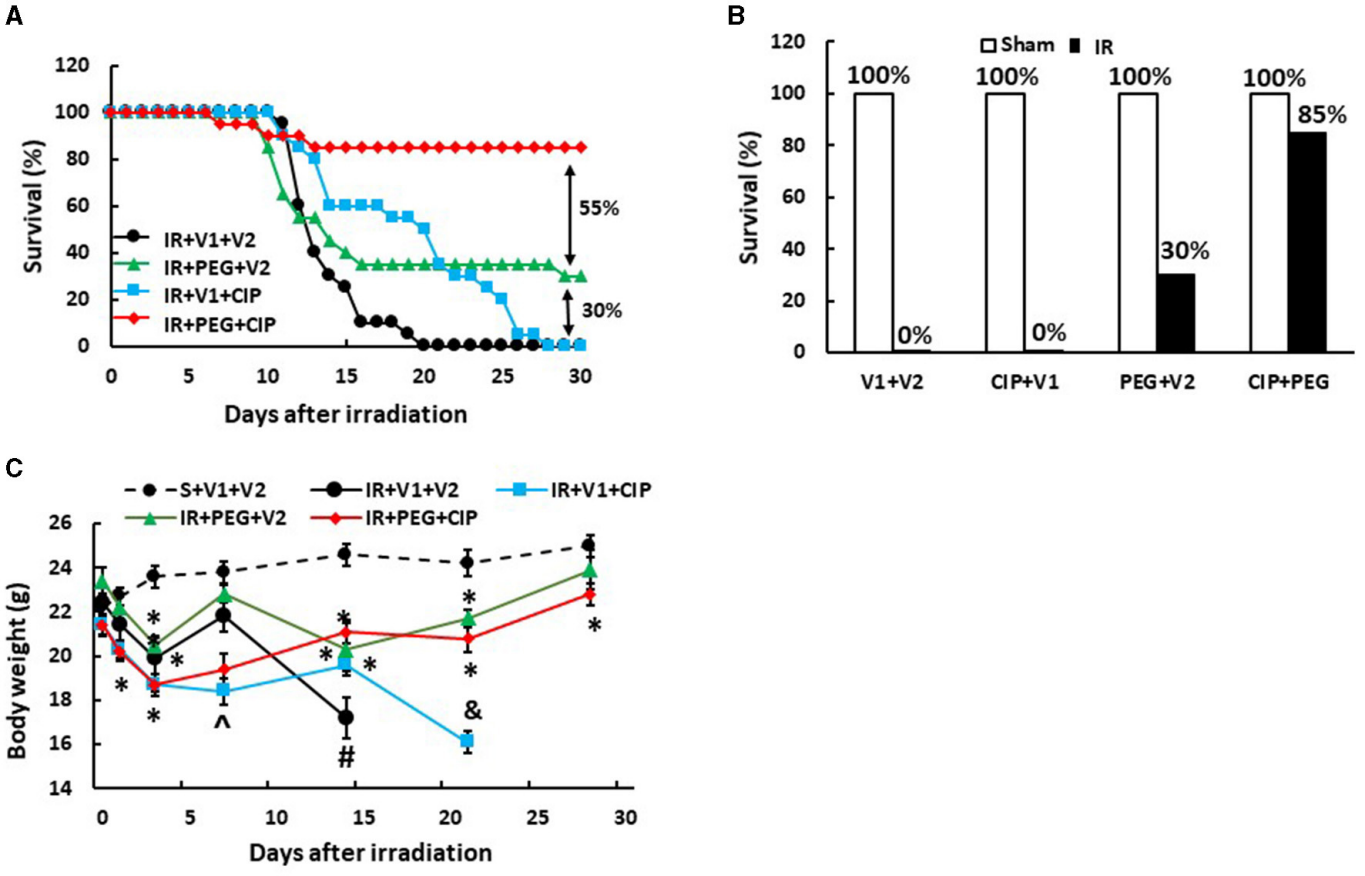
CIP enhances PEG efficacy on 30-day survival and mitigates body weight loss after IR. Animals were treated with CIP alone, PEG alone, or a combination of CIP + PEG. Survival was monitored daily for 30 days and body weight was measured on days 1, 3, 7, 14, 21, and 28. All sham-treated mice survived over 30 days. **(A)** Daily percent of the 30-day survival from each group is presented. **(B)** The end 30-day percent survival from each group is presented. **(C)** Body weights are shown. *N* = 20 per group. Data are presented as mean ± SEM. ^*^p < 0.05 vs. S + V1 + V2; ^∧^*p* < 0.05 vs. Sham + V1 + V2, RI + V1 + V2 and RI + PEG + CIP; ^#^*p* < 0.05 vs. Sham + V1 + V2, IR + V1 + CIP, and IR + PEG + CIP; ^&^*p* < 0.05 vs. Sham + V1 + V2, IR + PEG + V2, and IR + PEG + CIP. S, Sham; IR, ionizing radiation at 9.5 Gy; V1, vehicle for pegylated-G-CSF; V2, vehicle for Ciprofloxacin; CIP, Ciprofloxacin; PEG, pegylated G-CSF.

Our laboratory previously reported that treatment with PEG resulted in 25–35% survival improvement over the vehicle-treated mice after 9.5 Gy IR (10, 11), a dose that we have previously used to test drug efficacy (10, 11, 16–18) and those results were confirmed here. In order to enhance PEG's efficacy on survival, CIP was orally administered 2 h after irradiation but 22 h before the first injection with PEG and daily after for 21 days and induced an 85% survival on day 30, while CIP treatment alone and PEG treatment alone induced 0% and 30% improved survival, respectively, as shown on Figures 1A, B, suggesting the presence of a CIP enhancement. All sham-treated mice survived over 30 days. Furthermore, the surviving mice did not display any brain hemorrhages (data not shown). Figure 1C shows that CIP + PEG combined therapy significantly mitigated the IR-induced body weight loss on days 14, 21, and 28. IR mice treated with vehicle alone or CIP alone did not mitigate the body weight loss.

### 3.2 CIP therapy with PEG mitigates hemorrhagic lesions on brain surfaces after IR

**Figure 2:**
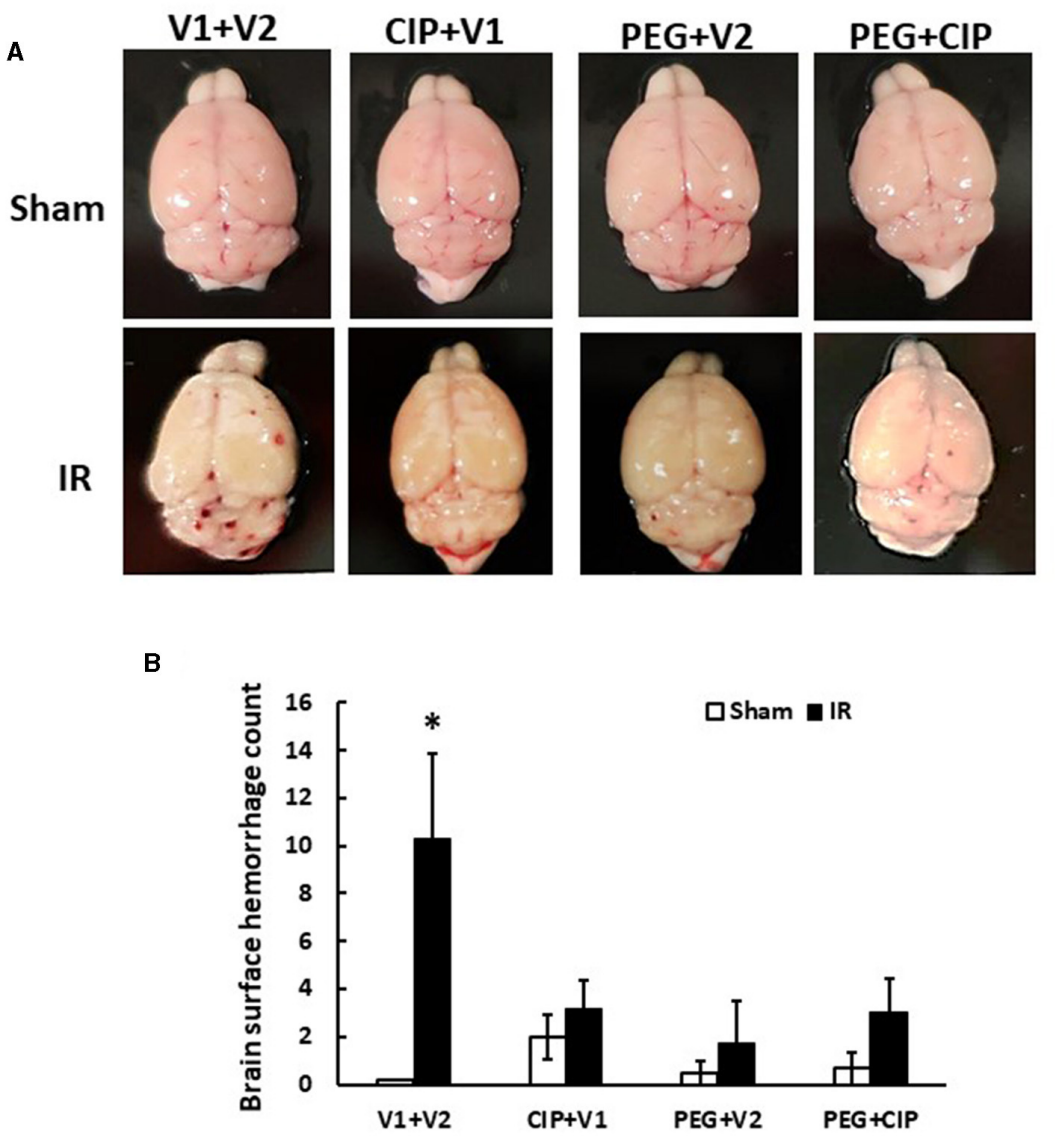
CIP therapy with pegylated G-CSF mitigates hemorrhagic lesions on brain surfaces on day 15 after IR. Animals were treated with CIP alone, PEG alone, or a combination of CIP + PEG. Brains were collected on day 15 post-irradiation. *N* = 5–6 per group. Data are presented as mean ± SEM. **(A)** Representative images of brains from each group. **(B)** Brain surface hemorrhage counts. *p < 0.05 vs. respective sham group. IR, ionizing radiation at 9.5 Gy; V1, vehicle for pegylated-G-CSF; V2, vehicle for Ciprofloxacin; CIP, Ciprofloxacin; PEG, pegylated G-CSF.

Gross pathology assessments of skulls and brains showed no observable hemorrhagic lesions after Sham treatment (data not shown). Likewise, brains collected on days 2, 4, and 9 after IR also did not reveal surface and intracranial hemorrhage (data not shown). However, as shown in Figure 2, brains collected from IR mice displayed hemorrhages appearing on the surfaces of cerebrum and cerebellum, with many hemorrhage lesions shown on the cerebellum on day 15 after IR. Treatment with CIP alone, PEG alone, or the combination of these two drugs significantly inhibited total brain surface hemorrhage lesions (Figure 2).

### 3.3 CIP therapy with PEG mitigates intracranial hemorrhagic lesions after IR

**Figure 3:**
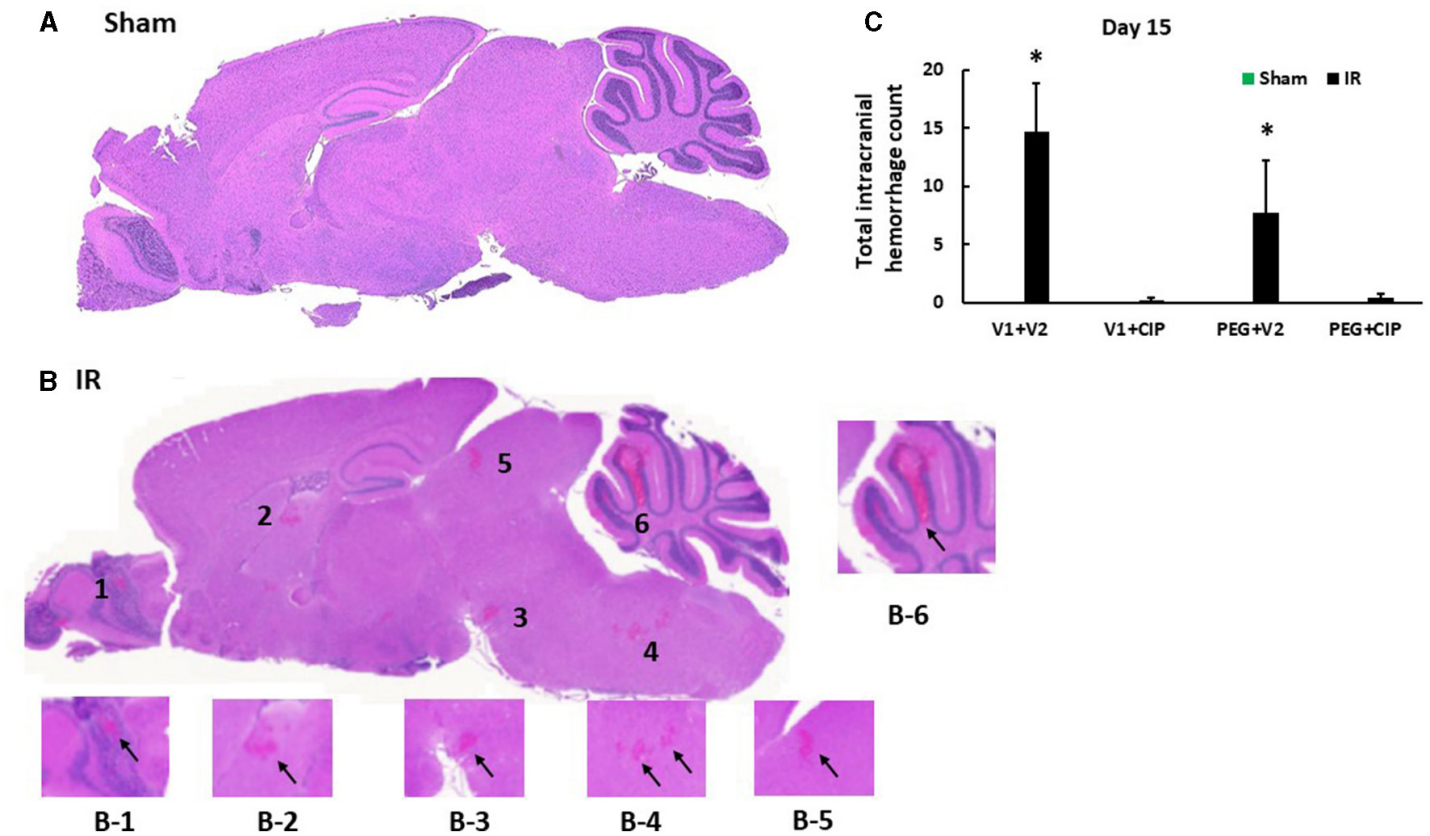
CIP therapy with PEG mitigates intracranial hemorrhagic lesions after IR. Animals were treated with CIP alone, PEG alone, or combination of CIP + PEG. Brains were collected on day 15 post-irradiation. *N* = 5–6 per group. Data are presented as mean ± SEM. **(A)** Representative sham brain histopathology image was presented. **(B)** The hemorrhages are indicated by the black arrows in each magnified area as labeled B1-B6. **(C)** Total intracranial hemorrhage count from each group was determined. *p < 0.05 vs. respective sham group. IR, ionizing radiation at 9.5 Gy; V1, vehicle for pegylated-G-CSF; V2, vehicle for Ciprofloxacin; CIP, Ciprofloxacin; PEG, pegylated G-CSF.

Our previous report showed the presence of hemorrhage in brains after IR followed by inflicted burn trauma (16). To evaluate the presence of intracranial hemorrhage after IR herein, histological slides with H & E staining were made. As shown in Figure 3A, IR made the brain tissues more granular than the shame-treated brain. IR induced intracranial bleeding lesions throughout neurons of olfactory lobe, forebrain, midbrain, hindbrain, cerebellum, pons, and medulla oblongata (Table 2 and Figure 3B). CIP alone and CIP + PEG fully inhibited the lesions whereas PEG alone did not (Table 2, Figure 3C).

**Table 2:** Intracranial hemorrhage counts via histopathological examination post-IR (*N* = 5–6 per group).

**Day 15**	**Olfactory Bulb**		**Forebrain**	**Midbrain**			
	**Epithelium**	**Neuron**			**Cerebellum**	**Pons**	**Medulla Oblongata**
Sham + V1 + V2	0	0	0	0	0	0	0
IR + V1 + V2	0	3.17 ±0.6	1.5 ± 0.62	3.5 ± 1.69	1.67 ± 0.8	2.83 ± 2.45	2 ± 2
Sham + V1 + CIP	0	0	0	0	0	0	0
IR + V1 + CIP	0	0	0	0	0.2 ± 0.2	0	0
Sham + PEG + V2	0	0	0	0	0	0	0
IR + PEG + V2	0	3 ± 3	1.5 ± 0.87	1.5 ± 0.96	0.5 ± 0.5	1.25 ± 1.25	0
Sham + PEG + CIP	0	0	0	0	0	0	0
IR + PEG + CIP	0	0.33 ± 0.21	0	0	0	0.17 ± 0.17	0

IR, ionizing radiation at 9.5 Gy; V1, vehicle 1 for pegylated G-CSF; V2, vehicle 2 for Ciprofloxacin; CIP, Ciprofloxacin; PEG, pegylated G-CSF.

**Figure 4:**
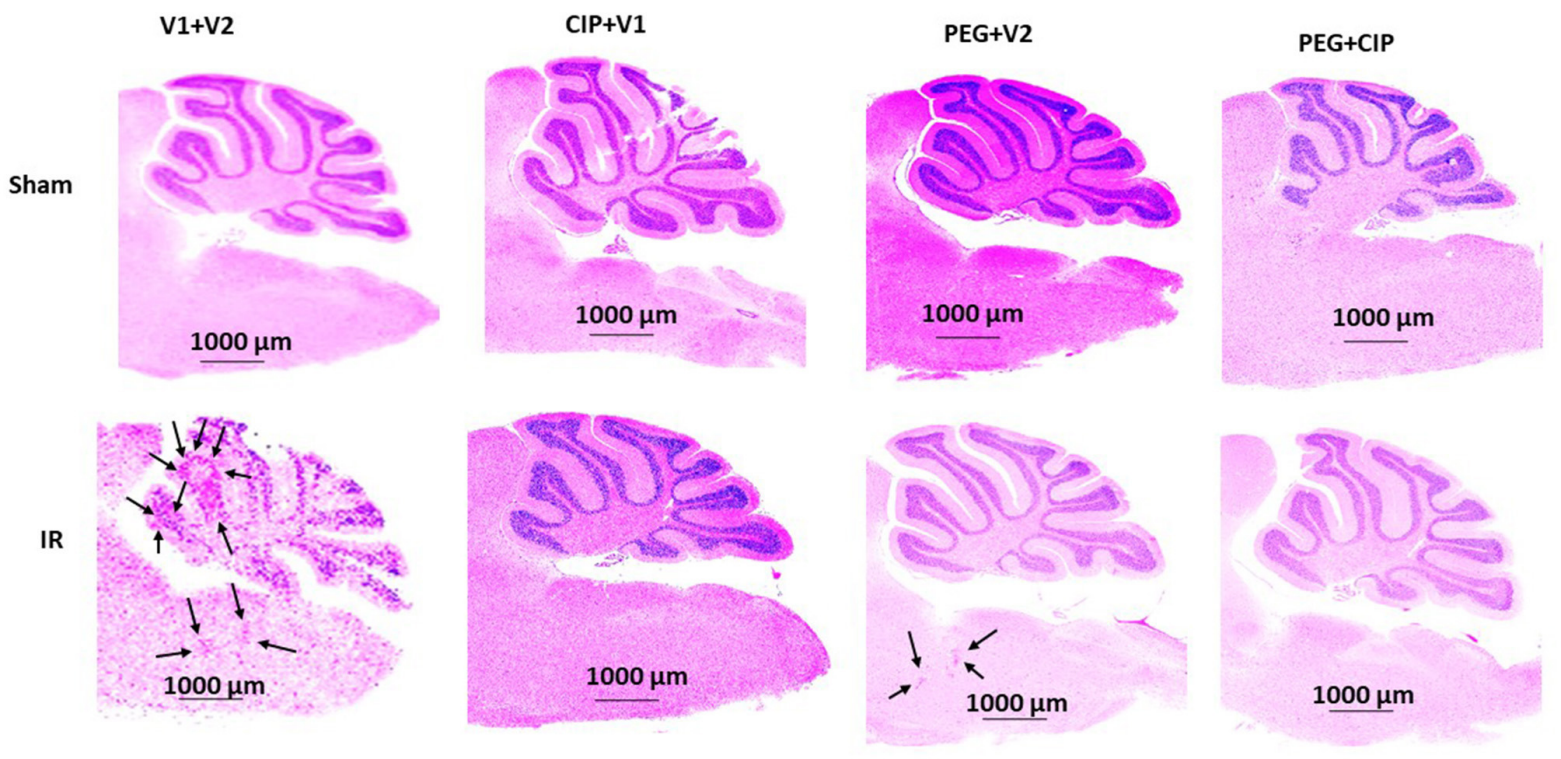
Representative images of cerebellum/pons/medulla oblongata from each group are presented. Animals were treated with CIP alone, PEG alone, or combination of CIP + PEG. Brains were collected on day 15 post-irradiation. *N* = 5–6 per group. IR, ionizing radiation at 9.5 Gy; V1, vehicle for pegylated-G-CSF; V2, vehicle for Ciprofloxacin; CIP, Ciprofloxacin; PEG, pegylated G-CSF.

Because hemorrhagic lesions were predominant in the cerebellum, pons, and medulla oblongata, these areas (Figure 4) were collected for the following biochemical analysis including changes in tight junctions and blood-brain barrier.

### 3.4 CIP therapy with PEG does not alter IR-induced ICAM1 increases and makes no impacts on Claudin 2 and ZO-1 in cerebellum/pons/medulla oblongata

**Figure 5:**
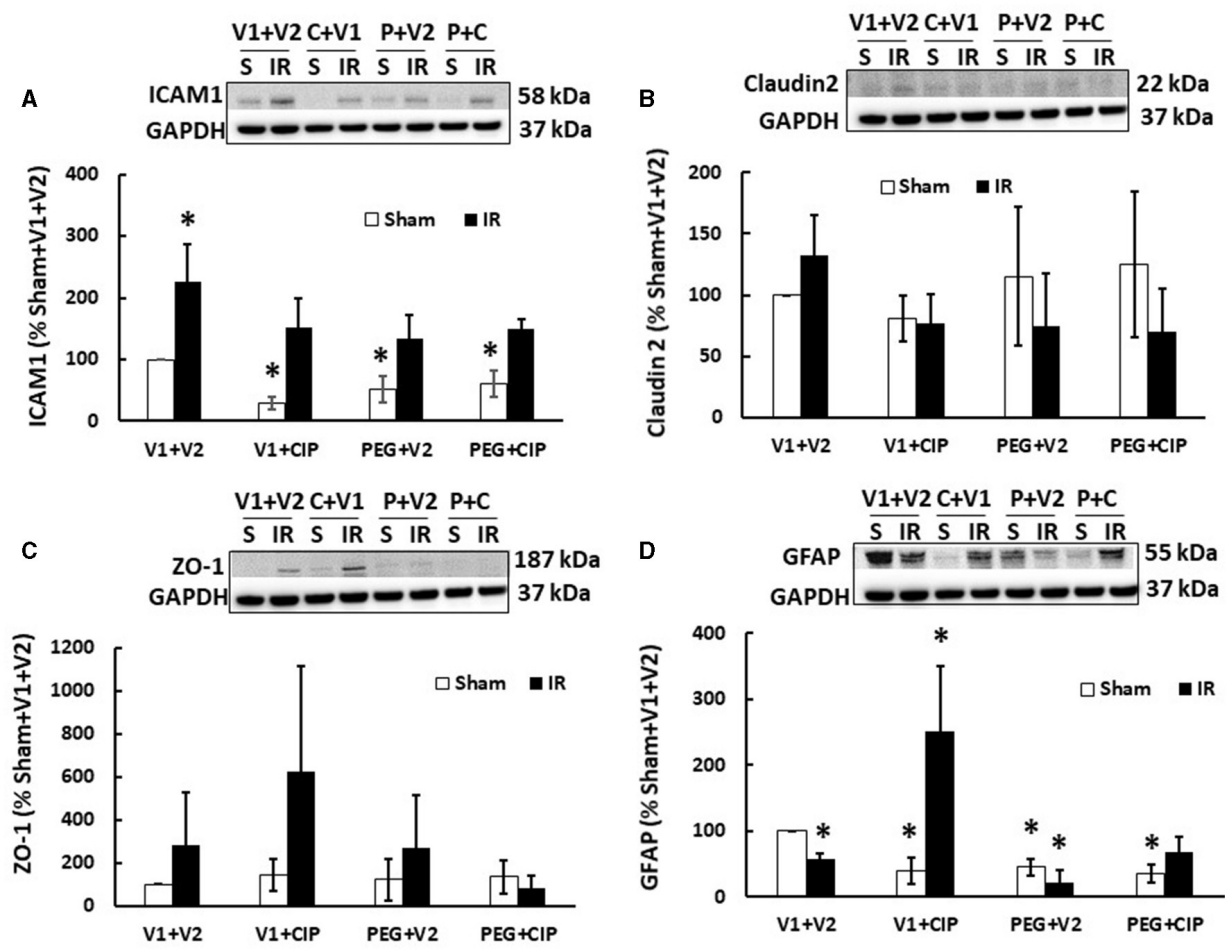
CIP therapy with PEG recovers the IR-induced GFAP decrease but not ICAM1 increases or tight junction in cerebellum/pons/medulla oblongata. Animals were treated with CIP alone, PEG alone, or combination of CIP + PEG. Brains were collected on day 15 post-irradiation. Lysate samples with cerebellum/pons/medulla oblongata were prepared to detect ICAM1 **(A)**, Claudian 2 **(B)**, ZO-1 **(C)**, and GFAP **(D)**. *N* = 5–6 per group. Data are presented as mean ± SEM. *p < 0.05 vs. sham + V1 + V2 group. IR, ionizing radiation at 9.5 Gy; V1, vehicle for pegylated-G-CSF; V2, vehicle for ciprofloxacin; C or CIP, ciprofloxacin; P or PEG, pegylated G-CSF.

Previously, we showed that irradiation decreased tight junctions in the ileum (19). Therefore, we investigated whether IR induced any changes in ICAM1 (as a biomarker for endothelium and blood-brain-barrier), Claudin 2 and ZO-1 (as biomarkers for brain tight junction) in the cerebellum/pons/medulla oblongata through western blot analysis. As shown in Figure 5, IR significantly increased ICAM1 (Figure 5A) but not Claudin 2 (Figure 5B) and ZO-1 (Figure 5C). CIP alone, PEG alone, or PEG + CIP decreased the ICAM1 baseline in non-irradiated animals, but they enabled to recover ICAM1 to the baseline. In contrast, the single or combined therapy did not alter either Claudin 2 (Figure 5B) or ZO-1 (Figure 5C) in non-irradiated or irradiated animals.

### 3.5 CIP therapy with PEG recovers radiation-induced glia fibrinogen acidic protein reduction in cerebellum/pons/medulla oblongata

Radiation increases GFAP in serum (20). Herein, we demonstrated that IR significantly decreased GFAP levels in lysate samples of mice treated with vehicle (Figure 5D), CIP therapy alone recovered GFAP and further increased it; PEG + CIP only recovered GFAP but did not further increase it. Unlike CIP, PEG alone decreased GFAP, suggesting PEG might have antagonized CIP exerting its action on GFAP, when PEG + CIP combined treatment was given. It should be noted that CIP alone, PEG alone or PEG + CIP declined the GFAP baselines, compared to the GFAP levels in sham + V1 + V2 group.

### 3.6 Megakaryocyte counts in sternums after IR

**Figure 6:**
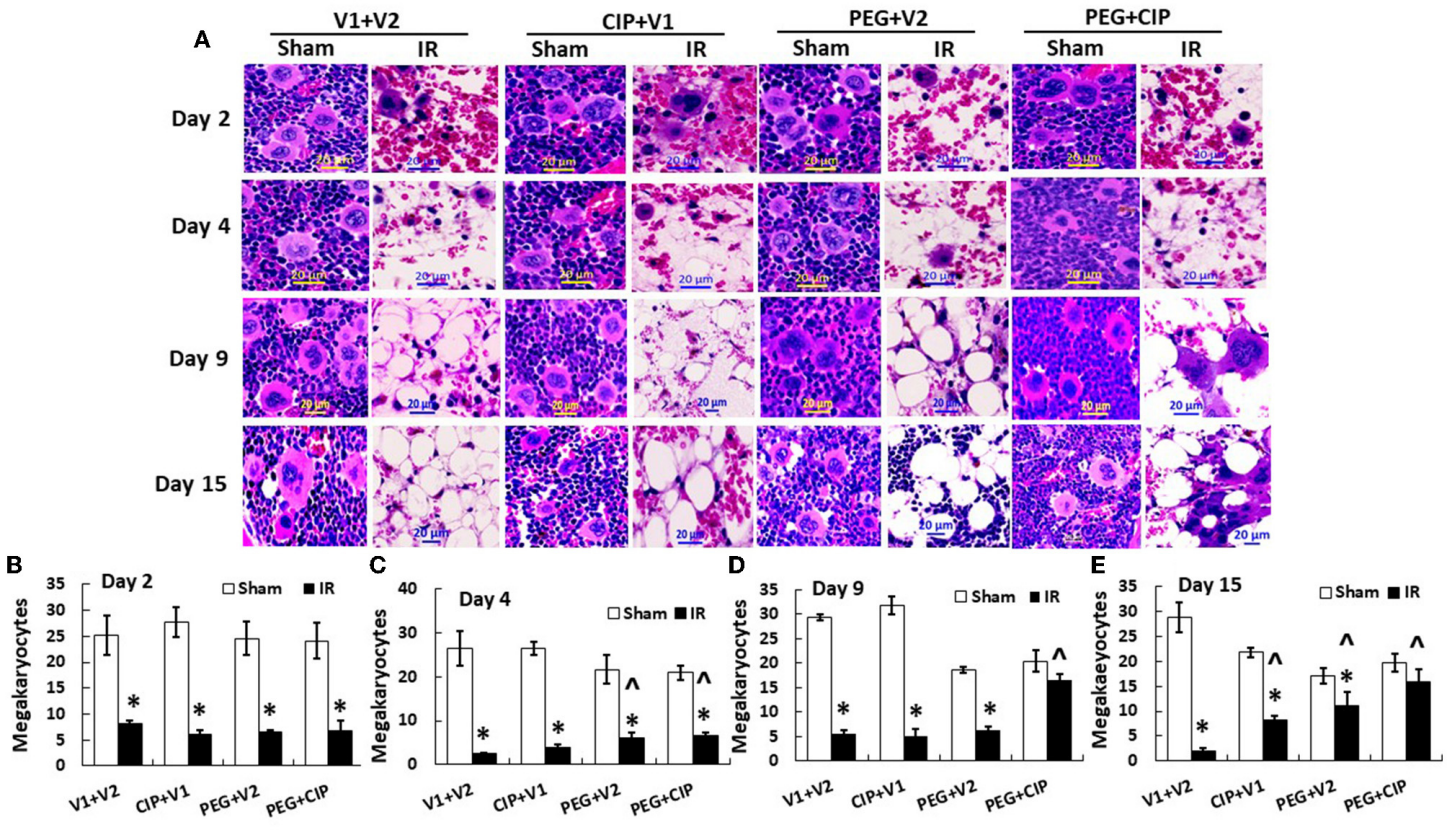
**(A–E)** CIP therapy with PEG recovers megakaryocyte counts after IR. Animals were treated with CIP alone, PEG alone, or a combination of CIP + PEG. Sternums were collected on days 2, 4, 9 and 15 post-IR for counting megakaryocytes in each group. *N* = 5–6 per group. Data are presented as mean ± SEM. *p < 0.05 vs. respective sham group. ^∧^*p* < 0.05 vs. IR + V1 + V2; IR, ionizing radiation at 9.5 Gy; V1, vehicle for pegylated-G-CSF; V2, vehicle for Ciprofloxacin; CIP, Ciprofloxacin; PEG, pegylated G-CSF.

IR decreases megakaryocyte counts (18). Since platelets are derived from megakaryocytes, in this time-course study of sternums, bone marrows from sternums were measured. As shown on Figure 6, IR decreased megakaryocytes on day 2, continued to decrease on day 4, and megakaryocytes remained low on days 9 and 15. CIP alone recovered megakaryocytes on day 15. In contrast, PEG began to recover megakaryocytes on day 4 and day 15. PEG + CIP recovered megakaryocytes on days 4, 9, and 15.

### 3.7 CIP therapy with PEG partially recovers platelet counts caused by IR

**Figure 7:**
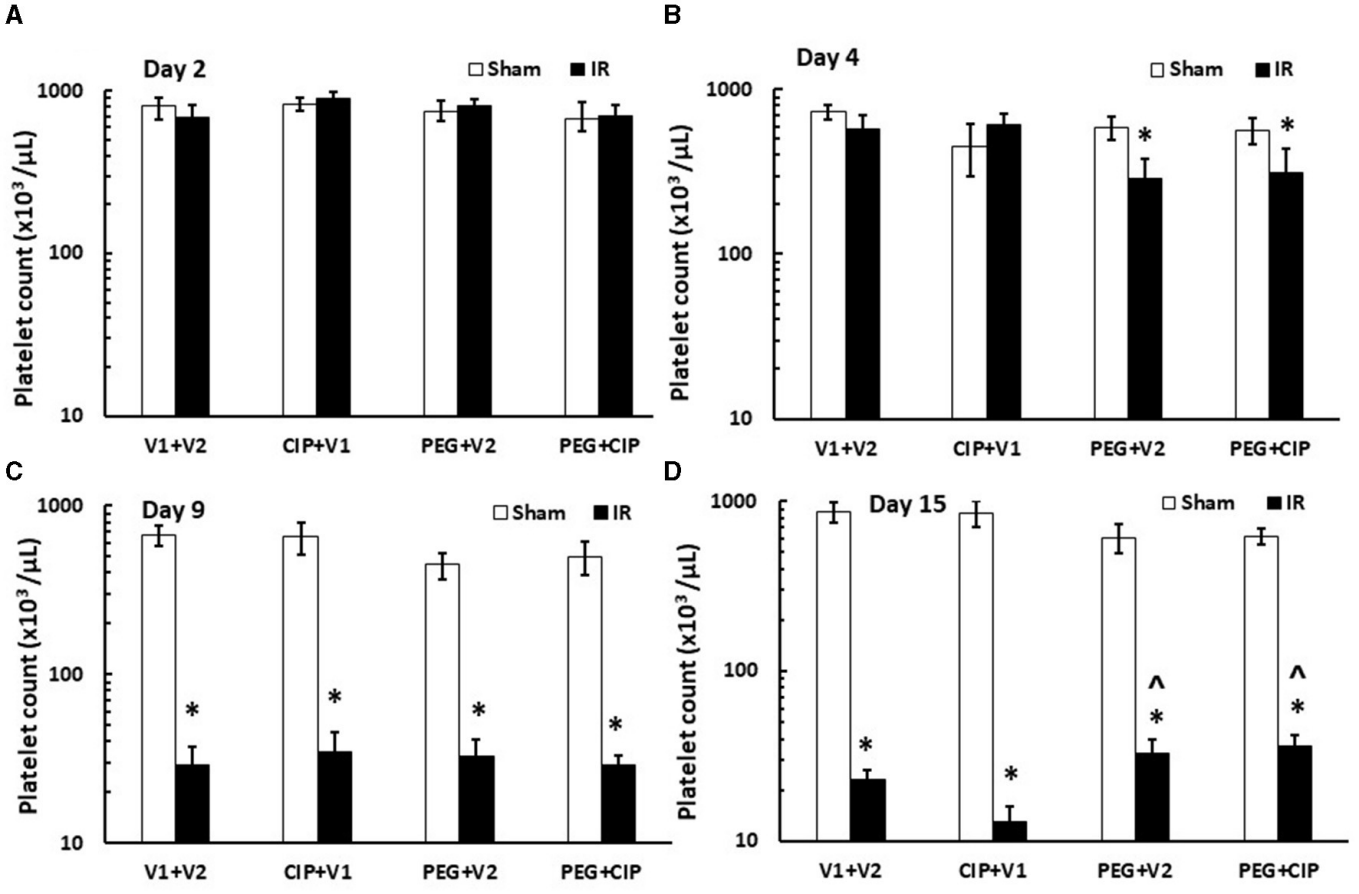
**(A–D)** CIP therapy with PEG partially recovers platelet counts caused by IR. Animals were treated with CIP alone, PEG alone, or a combination of CIP + PEG. Blood was collected on days 2, 4, 9, and 15 post-IR for measuring platelet counts in each group. *N* = 5–6 per group. Data are presented as mean ± SEM. *p < 0.05 vs. Sham + V1 + V2 group; ^∧^*p* < 0.05 vs. IR + V1 + V2 group. IR, ionizing radiation at 9.5 Gy; V1, vehicle for pegylated-G-CSF; V2, vehicle for Ciprofloxacin; CIP, Ciprofloxacin; PEG, pegylated G-CSF.

IR induces platelet depletion on day 7 post irradiation (19, 21). In this time-course study, IR did not decrease platelet counts on days 2 and 4, but significantly decreased platelet counts on day 9 and day 15 (*p* < 0.05). On day 15, PEG alone or PEG + CIP combined therapy began to recover platelet counts (Figure 7) that was co-incidental with the observation of brain hemorrhage (Figure 3B).

### 3.8 CIP therapy with PEG inhibits IR-induced increases in serum IL-18

**Figure 8:**
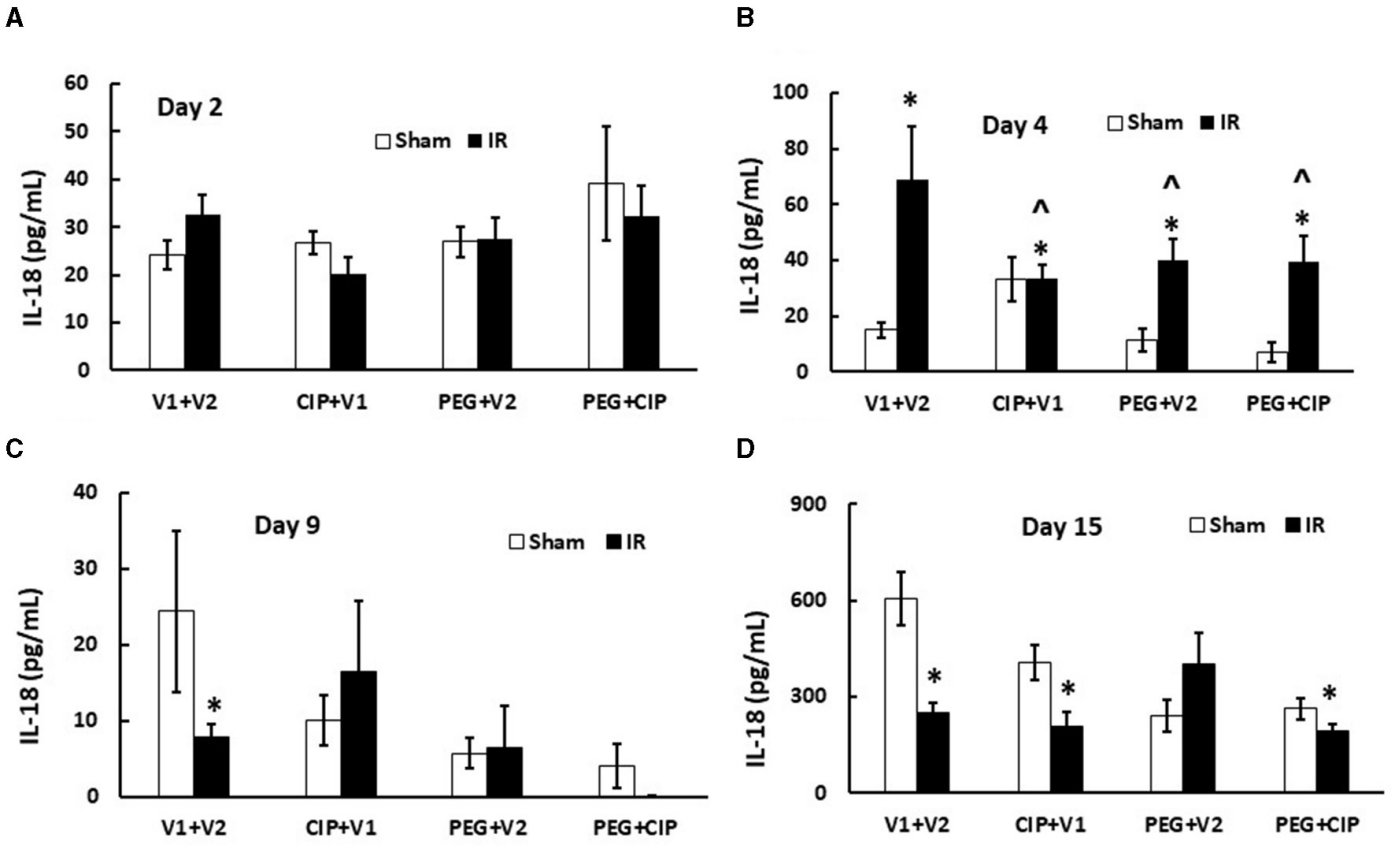
**(A–D)** CIP therapy with PEG inhibits IR-induced increases in serum IL-18. Animals were treated with CIP alone, PEG alone, or combination of CIP + PEG. Blood was collected on days 2, 4, 9, and 15 post-IR for measuring IL-18 levels in each group. *N* = 5–6 per group. Data are presented as mean ± SEM. *p < 0.05 vs. Sham + V1 + V2 group; ^∧^*p* < 0.05 vs. IR + V1 + V2 group. IR, ionizing radiation at 9.5 Gy; V1, vehicle for pegylated-G-CSF; V2, vehicle for Ciprofloxacin; CIP, Ciprofloxacin; PEG, pegylated G-CSF.

IR increases cytokine/chemokines in blood and tissues (7, 17, 19, 22–25). We found similar results in cerebellum/pons/medulla oblongata after IR. IR increased IL-6, KC, Eotaxin, G-CSF, MIP-2, MCP-1, and MIP-1α, but decreased IL-18 in cerebellum lysates on days 12–18 (7). Therefore, IL-18 levels in serum on days 2, 4, 9, and 15 were measured. As shown on Figure 8, IR began to significantly increase IL-18 levels in serum on day 4 and the increase disappeared on days 9 and 15. CIP alone, PEG alone, or combination of 2 drugs significantly mitigated the IL-18 increases on day 4.

### 3.9 CIP therapy with PEG mitigates IR-induced increases in complement protein 3 (C3) in circulation

**Figure 9:**
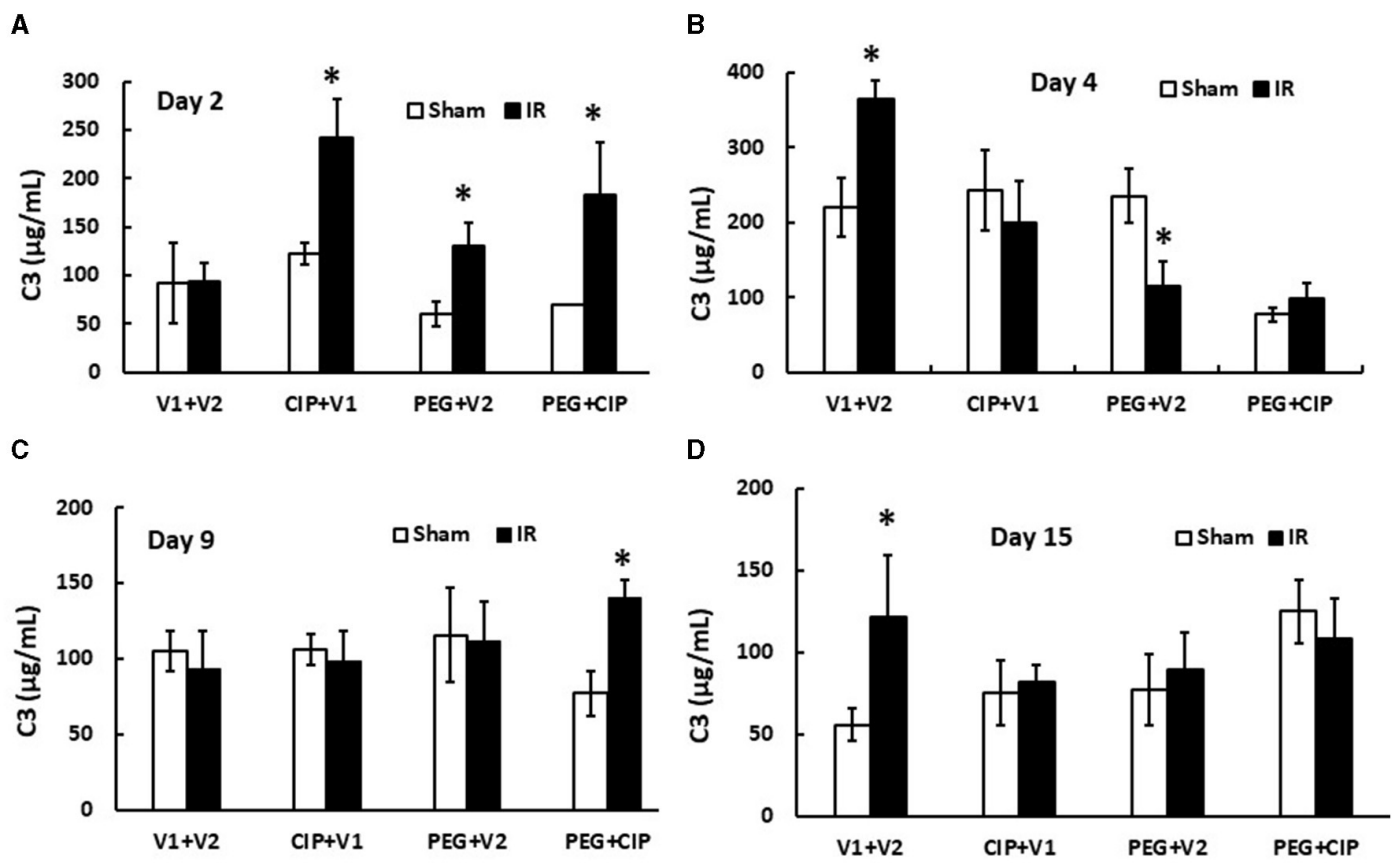
**(A–D)** CIP therapy with PEG mitigates IR-induced increases in complement protein 3 (C3) in circulation. Animals were treated with CIP alone, PEG alone, or combination of CIP + PEG. Blood was collected at different time points post-irradiation for measuring C3 levels in each group. *N* = 5–6 per group. Data are presented as mean ± SEM. *p < 0.05 vs. Sham + V1 + V2 group. IR, ionizing radiation at 9.5 Gy; V1, vehicle for pegylated-G-CSF; V2, vehicle for Ciprofloxacin; CIP, Ciprofloxacin; PEG, pegylated G-CSF.

IR increases complement protein C3 in serum (26). Therefore, C3 levels in serum on days 2, 4, 9, and 15 were measured. IR did not increase C3 on day 2, but did significantly on day 4. The C3 level returned to the baseline on day 9 but increased again on day 15 coinciding with the appearance of brain hemorrhages. On day 2, CIP, PEG, or the combination significantly increased C3 in serum from IR mice. On day 4 and day 15, CIP, PEG, or the combination fully mitigated C3 levels in IR mice. On day 9, the combined therapy but not CIP or PEG alone increased C3 in irradiated mice (Figure 9). The results suggest a direct correlation between C3 and brain hemorrhage after irradiation.

### 3.10 CIP therapy with PEG mitigates IR-induced increases in p53 activation

**Figure 10:**
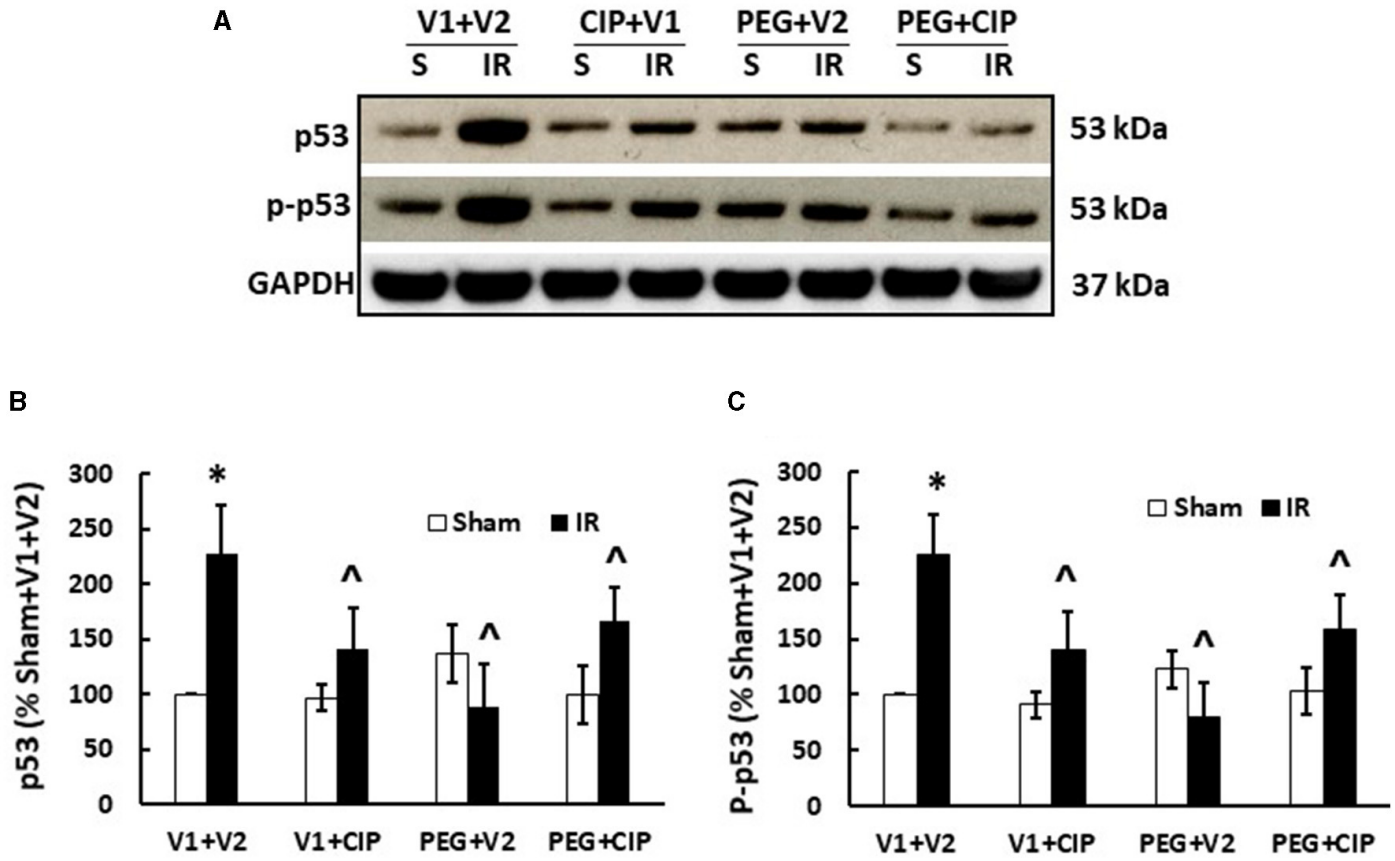
CIP therapy with PEG recovers IR-induced increases in p53 and phosphorylated p53 in cerebellum/pons/medulla oblongata. Animals were treated with CIP alone, PEG alone, or combination of CIP + PEG. Brains were collected on day 15 post-IR. Lysate samples with cerebellum/pons/medulla oblongata were prepared to detect p53 and p-p53. **(A)** Representative gel images were presented, **(B)** p53, **(C)** phosphorylated p53. *N* = 5–6 per group. Data are presented as mean ± SEM. *p < 0.05 vs. respective sham group. ^∧^*p* < 0.05 vs. IR + V1 + V2. S: Sham; IR, ionizing radiation at 9.5 Gy; V1, vehicle for pegylated-G-CSF; V2, vehicle for Ciprofloxacin; CIP, Ciprofloxacin; PEG, pegylated G-CSF.

IR increases p53 activation (7, 20, 27). Therefore, p53 activation in brain was studied on day 15. As expected, IR significantly increased both p53 (Figures 10A, B) and phosphorylated p53 (Figures 10A, C). CIP mitigated these elevations. PEG and PEG + CIP fully inhibited them, suggesting p53-mediated cell death may occur subsequently. However, IR was reported to increase p16 and p21 (biomarkers for cell senescence) in brains on day 30 (18). Therefore, we measured these two proteins in cerebellum/pons/medulla oblongata and found out they were undetectable on day 15 (data not shown).

## 4 Discussion

In this report, we provide evidence that in B6D2F1/J mice, brain hemorrhage was observed in IR mice at 15 days after IR at 9.5 Gy. Although this dose was the LD50/30 in our previous reports, in this experiment, this radiation dose resulted in 100 % mortality in IR mice treated with vehicles. The higher killing effect might be caused by the newly calculated radiation map that was used at that time. Generally, the LD_50/30_ is based on average data from several experiments over several years, which can vary in actual mortality. Since, this newly calculated radiation map was used after our experiment, 9.5 Gy had been observed to be LD_75/30_ (12) and LD_70/30_ (20). Similar variation has been found with other strains of mice. We indeed do not exactly know what leads to this. Therefore, it is under investigation.

Regardless of the variations mentioned above, CIP and PEG combined therapy after IR effectively increased survival above the vehicle treatment by 85%, which was above PEG alone by 55%. The PEG alone treatment resulted in 30% above the vehicle group, which was in consistency with the findings reported in the literature (8, 9, 12). The combined therapy also successfully mitigated occurrence of the IR-induced weight loss and brain hemorrhage.

As shown in the previous report (7), the IR-induced brain hemorrhage was generally distributed on the olfactory lobe, forebrain, midbrain, hindbrain, cerebellum, pons and medulla oblongata. Among these, the cerebellum, pons and medulla oblongata displayed more bleeding than other brain areas, both on the brain surface and internally, suggesting that movement, cardiovascular regulation and respiratory regulation would be immensely impacted by the lesions. To our surprise, our results show that 9.5 Gy ^60^Co-gamma photon radiation is sufficient to cause brain hemorrhage, which breaks the doctrine that brain is insensitive to radiation unless exposed to very high dose such as 30–51 Gy (28).

Either total or partial body radiation exposure results in damage of microvascular networks, which is one of the most important outcomes of acute radiation sickness (1, 4, 29–31). IR concurrently induces the massive release of numerous reactive factors, coagulopathy, suppression of vascular growth factors and vascular remodeling, and the complication of endothelial injury-associated peripheral perfusion (32, 33). The microvascular barriers (composed of vascular endothelial cells, the basement membrane and pericytes) sustain circulatory homeostasis. Therefore, the impact of endothelium impairment becomes long-lasting, from an acute phase to a delayed phase, and thereafter, to a prolonged phase (4, 5, 20, 32, 33). These effects of interstitial hemorrhage, cell hypoxia, and cell necrosis are life-threatening and represent a great challenge; not only in the development of countermeasures against radiological/nuclear accidents, but also because they can complicate outcomes in radiation therapy (4, 34–36). Our data showed that IR significantly increased the ICAM1 levels in the brain lysates, while CIP alone, PEG alone, or CIP + PEG all failed to decrease it, suggesting the IR-induced th1-e endothelium injury was persistent. Our data were consistent with the data observed from cerebral cortex in an experiment with the whole brains of male C57BL/6J mice that were exposed to 20 Gy IR and ICAM1 was measured 24 h and 48 h post-IR (37).

IR reduced GFAP and CIP alone and CIP + PEG, but not PEG alone, enabled the recovery of GFAP, which is mainly produced by astrocytes (38). Thus, the IR-induced GFAP reduction may have contributed to the IR-induced BBB injury and was reversed by either a CIP or a combined PEG + CIP therapy. Other laboratories reported IR increased GFAP (39, 40). One laboratory exposed the whole brain of male Sprague-Dawley rats to 15 Gy X-ray linear accelerator and measured GFAP from the whole brain 6 h and 24 h post-IR (40). The other laboratory exposed the whole brain of male BALB/C mice to 10 Gy X-ray and measured GFAP from the whole brain on days 1, 7, 30, 90, 180 post-IR (39). The discrepancies between their studies and our study were: (i) that we had female B6D2F1 mice, whereas they had male Sprague-Dawley rats (40) or male BALB/C mice (39); (ii) that we measured GFAP from cerebellum/pons/medulla oblongata, whereas they did GFAP from the whole brain (39, 40); (iii) that our time point was day 15, whereas theirs was either at 6 h and 24 h post-IR (40), or at 1, 7, 30, 90, and 180 days post-IR (39); and (iv) that we exposed whole-body mice at 9.5 Gy Co-60 γ-photon radiation, whereas they exposed the brain only at 25 Gy X-ray (40) or 10 Gy X-ray (39). Collectively speaking, different biological sex, strains, radiation sources, time-points studied, and parts of brain measured may explain why our results were different from theirs.

Unlike ileum, IR did not alter Claudin 2 and ZO-1 levels in the brain, suggesting the cell tight junctions was not damaged by IR and that the brain tight junctions are less sensitive to IR compared to that observed in the ileum (18).

IR reduced megakaryocyte counts in bone marrow on day 2 post-IR, thereby, perhaps leading to platelet depletion on day 4 and continued to deplete them on day 15. Although PEG alone and CIP + PEG mitigated the megakaryocyte reduction on days 4 and 15 and days 4–15, respectively (Figure 6), the recovery of circulating platelet counts was not seen until day 15 (Figure 7), suggesting there may be a time-lag between the megakaryocyte production in bone marrow and platelet replenishment in circulation. This platelet replenishment is important for inhibiting brain hemorrhage. This observation was consistent with our earlier publication (7). Megakaryocyte sizes are about 100 μm in diameter, whereas platelet sizes are about 2 μm in dimeter. Thrombopoietin (TPO) production by the liver will be stimulated by decreases in platelet counts in peripheral blood. Consequently, it will result in an increased number of megakaryocytes in bone marrow. It takes about 5 days in humans and 2–3 days in rodents for megakaryocytes to complete polyploidization, mature, and release platelets (41–43). Once released into the bloodstream, human platelets survive 7–10 days, whereas rodent platelets in peripheral blood survive 4–5 days (44–46). The osteoblastic niche provides an environment that allows megakaryocytes to mature and develop, while the vascular niche enhances proplatelet formation (47). Therefore, the possibility of CIP and PEG combined therapy stimulating the vascular niche cannot be excluded. The FDA-approved Nplate (Romiplostim) which is a thrombopoietin receptor agonist that can activate production of thrombocytes (48) could also be effective along this line of thinking and should be explored.

IR induces GI-ARS and causes systemic bacterial infection (21). CIP is known to kill Gram-negative bacteria (49). Therefore, the microbiome in fecal pellets collected on days 2, 4, 9, and 15 in this study was examined. Their α/β diversities and volcano analysis are under investigation. Whether CIP restores the microorganisms in the GI and whether this microbiome restoration contributes to CIP enhancement on brain repair and survival are underway.

IR increased cytokines/chemokines in circulation including IL-18 (18, 24), whereas IR decreased IL-18 in brain tissue (7). Although CIP alone, PEG alone, and CIP + PEG effectively inhibited IL-18 in serum, the results seem to suggest that IL-18 may not be associated with the IR-induced brain hemorrhage.

In contrast to IL-18, complement protein C3 is believed to play a key role in the IR-induced brain synapse homeostasis. C3 is known to be involved in neuronal synapse pruning by microglia (50, 51) through the classical complement protein cascade (52), suggesting that the IR-induced complement C3 increases are detrimental to CNS synapses. CIP, PEG, and CIP + PEG were able to fully inhibit the increases (Figure 8), suggesting that a single therapy or a combined therapy may potentially alleviate the brain impairment caused by IR, and may be efficacious in preserving brain function and integrity. Furthermore, CIP alone, PEG alone, and PEG + CIP successfully attenuated p53 activation (Figure 10). However, IR was known to reduce AKT activation in conjunction with an elevated MAPK activation in cerebellum/pons/medulla oblongata (7). Whether CIP + PEG mitigation of brain hemorrhage and impairment is mediated by recovering AKT activation and inhibiting MAPK activation should be further explored. Additionally, IR significantly increased miR-34a levels in ileum (1) that was triggered by the IR-induced p53 activation (53, 54). The possibility of miR-34a involvement in brain hemorrhage and impairment cannot be ruled out. It should be noted that IR increased IL-6 levels in cerebellum/pons/medulla oblongata (7) and increased IL-6 expression is known to lead to astrocyte senescence (55). Herein, p16 and p21 in cerebellum/pons/medulla oblongata lysates on day 15 post-IR were not detected. This discrepancy can be due to a different part of brain investigated and their time-point of brain collected was on day 30 post-IR (20) vs. day 15 in this report.

Herein, our data show that CIP alone and CIP + PEG after IR effectively (a) recovered megakaryocytes in bone marrow of sternums, (b) recovered GFAP and further increases in GFAP in CIP-treated brains, (c) inhibited C3 and p53 activation, and (d) inhibited intracranial hemorrhage. On the other hand, PEG alone increased circulating platelets but not GFAP recovery as well as not mitigating intracranial hemorrhage, suggesting that CIP + PEG enhancement on 30-day survival compared to PEG alone after IR may be partially contributed by increases in GFAP levels and decreases in p53 activation in brain, which is also participated in mitigating the IR-induced intracranial brain hemorrhage. Data to demonstrate CIP contribution to mitigating the IR-induced GI injury are on the way in our laboratory.

In summary, CIP enhanced PEG efficacy in survival and reduced weight loss after IR. IR significantly increased brain hemorrhage and these lesions were significantly mitigated by CIP therapy or CIP + PEG combined therapy. The IR-induced brain hemorrhage seemed to be mediated by platelet depletion, GFAP level reduction, and increases in complement protein C3 and p53 activation but was not associated with the IL-18 increases. CIP alone, PEG alone and CIP + PEG effectively recovered megakaryocytes in bone marrow of sternums, but CIP recovered GFAP and further increased GFAP level in brains, in conjunction with inhibition of C3 and p53 activation and intracranial hemorrhage. These results suggest that CIP + PEG combined therapy is potentially efficacious for treating the IR-induced brain hemorrhage, probably mediated by recovering GFAP and inhibiting increases in C3 and p53 activation. The study provides a new therapeutic approach for ARS by combining CIP with PEG and gives new insight into brain hemorrhage occurring after whole-body high dose radiation exposure.

## Data availability statement

The original contributions presented in the study are included in the article/supplementary material, further inquiries can be directed to the corresponding author.

## Ethics statement

The animal study was approved by the Uniformed Service University of the Health Sciences IACUC. The study was conducted in accordance with the local legislation and institutional requirements.

## Author contributions

JK: Conceptualization, Data curation, Formal analysis, Funding acquisition, Investigation, Methodology, Project administration, Resources, Supervision, Validation, Visualization, Writing – original draft, Writing – review & editing. GC: Data curation, Formal analysis, Investigation, Methodology, Writing – review & editing. MO: Data curation, Formal analysis, Investigation, Methodology, Supervision, Visualization, Writing – review & editing. MZ: Data curation, Formal analysis, Investigation, Methodology, Writing – review & editing. AW: Data curation, Formal analysis, Methodology, Writing – review & editing. FX: Methodology, Writing – review & editing. BL: Data curation, Formal analysis, Investigation, Methodology, Writing – review & editing. XL: Methodology, Writing – review & editing. LH: Data curation, Methodology, Writing – review & editing. SJ: Data curation, Formal analysis, Investigation, Methodology, Supervision, Writing – review & editing. MX: Investigation, Methodology, Resources, Supervision, Writing – review & editing.

## Author disclaimer

The views, opinions, and findings contained in this report are those of the authors and do not reflect official policy or positions of the Armed Forces Radiobiology Research Institute, the Uniformed Services University of the Health Sciences, the National Institute of Allergy and Infectious Diseases, the Department of Defense, or the United States government. The commercial products used in this report do not necessarily imply recommendation or endorsement by the Federal Government and do not imply that the products used are necessarily the best available for the purpose.
